# A Trauma-Informed Approach to the Medical History: Teaching Trauma-Informed Communication Skills to First-Year Medical and Dental Students

**DOI:** 10.15766/mep_2374-8265.11160

**Published:** 2021-06-07

**Authors:** Taylor Brown, Pooja K. Mehta, Sarah Berman, Katherine McDaniel, Caitlin Radford, Annie Lewis-O'Connor, Samara Grossman, Jennifer Potter, David A. Hirsh, Beverly Woo, David Krieger

**Affiliations:** 1 Fourth-Year Medical Student, Harvard Medical School; 2 Second-Year Resident, Department of Medicine, Brigham and Women's Hospital; 3 First-Year Resident, Department of Psychiatry, Cambridge Health Alliance; 4 Nurse Practitioner, Department of Medicine, Division of Women's Health, Brigham and Women's Hospital; Instructor, Harvard Medical School; 5 Social Worker, Department of Psychiatry, Brigham and Women's Hospital; 6 Professor, Department of Medicine, Harvard Medical School; Physician, Department of General Internal Medicine, Beth Israel Deaconess Medical Center; 7 The George E. Thibault Academy Associate Professor, Harvard Medical School; Physician, Department of Internal Medicine, Cambridge Health Alliance; 8 Associate Professor of Medicine, Harvard Medical School; Senior Physician, Department of Medicine, Brigham and Women's Hospital; 9 Clinical Instructor in Medicine, Harvard Medical School; Physician, Atrius Health

**Keywords:** Trauma, Trauma-Informed Care, Communication Skills, Intimate Partner Violence, Case-Based Learning, Flipped Classroom, Self-Assessment

## Abstract

**Introduction:**

Trauma is ubiquitous and associated with negative effects on physical and mental health. Trauma-informed care (TIC) is a framework for mitigating these health effects and improving patients’ engagement with medical care. Despite these clinical benefits, TIC is not routinely taught in undergraduate medical education.

**Methods:**

We designed a session for first-year medical and dental students to introduce TIC principles and their application in patient care. The session focused on screening for and inquiring about trauma and responding to disclosures of trauma. Using live patient interviews, small-group discussions, and case-based role-plays, the session offered expert instruction and hands-on practice. Students completed pre- and postsession surveys and a 5-month follow-up survey. Students reported their comfort with screening for trauma and responding to disclosures of trauma before and after the session and at 5 months following the session.

**Results:**

Of the 164 student participants, 76% completed surveys during the session, and 50% completed the follow-up survey. More than one-third (34%) of respondents reported having received at least one disclosure of trauma from a patient within the first 5 months of medical school. Students’ comfort with screening for trauma increased from 30% to 56%, and their comfort with responding to disclosure of trauma increased from 35% to 55%. These improvements persisted on reevaluation at 5 months.

**Discussion:**

We present a model for teaching trauma-informed communication skills to first-year medical and dental students. The intervention significantly increased students’ comfort level and self-reported clinical skills, and benefits persisted at 5 months.

## Educational Objectives

By the end of this activity, learners will be able to:

1. Define trauma and explain its prevalence and health impacts.
2. Describe the six principles of trauma-informed care (TIC) as defined by the Substance Abuse and Mental Health Services Administration.
3. Demonstrate a trauma-informed approach to the patient history.
4. Screen for and inquire about trauma, including intimate partner violence, using open-ended questions or a validated screening tool, and respond appropriately to disclosures of trauma using TIC principles.

## Introduction

Trauma is defined by the Substance Abuse and Mental Health Services Administration (SAMHSA) as “an event, series of events, or set of circumstances that is experienced by an individual as physically or emotionally harmful or life-threatening and that has lasting adverse effects on the individual's functioning and mental, physical, social, emotional, or spiritual well-being.”^1^ Traumatic events can include sexual, physical, and verbal abuse; natural disasters and accidents; war and terrorism; stigma and discrimination; and iatrogenic trauma, as well as other experiences.^2^ Trauma is ubiquitous. A review of 26 World Health Organization World Mental Health Surveys conducted in 24 countries of various income levels found that roughly 70% of respondents had experienced at least one traumatic event in their lifetime.^3^ A US study found almost 90% of adults reported at least one traumatic event in their lifetime as defined by the *Diagnostic and Statistical Manual of Mental Disorders-5.*^4^

A traumatic event may cause acute physical injuries with associated sequelae and poor long-term mental and physical health outcomes.^5^ Trauma can lead to the development of posttraumatic stress disorder, which has a lifetime prevalence of 6% for men and 13% for women.^4^ Trauma can overwhelm coping mechanisms, elevate or exacerbate stress, and alter health-seeking behaviors.^6^ Specific traumas that occur in childhood, known as adverse childhood experiences, exhibit a dose-response relationship with negative mental and physical health outcomes in adulthood.^7^ For example, adults with the highest levels of childhood trauma were found to be 12 times more likely to attempt suicide and twice as likely to suffer from cardiovascular disease.^7^ Exposure to trauma has been linked to higher rates of substance use, depression, anxiety, chronic pain, and cancer.^5,8^

Trauma-informed care (TIC) provides a framework for addressing and mitigating these health effects. TIC encourages health care providers, institutions, and systems to realize the impact of trauma; recognize the signs and symptoms of trauma; respond by integrating knowledge about trauma into policies, procedures, and practice; and seek to avoid retraumatization.^1^ TIC focuses on six key principles as defined by SAMHSA: safety (ensuring physical and psychological safety); trustworthiness and transparency (making decisions with transparency to build and maintain trust); peer support (promoting mutual support to aid in healing and recovery); collaboration and mutuality (leveling power differentials); empowerment, voice, and choice (ensuring autonomy and promoting individuals’ strengths); and cultural, historical, and gender issues (acknowledging and addressing the impact of historical trauma, overt discrimination, and implicit biases).^1^ Given their professional values and mission, health care providers and institutions must avoid adding to the trauma burden of their patients, providers, and trainees. They can advance this goal by adopting a universal, systematic, trauma-informed approach.^9^

In recent years, health care organizations have begun to incorporate TIC into clinical settings,^9^ including primary care,^10^ psychiatry,^11^ pediatrics,^12^ and dentistry.^13^ Within UME, trauma-informed medical education (TIME) has been proposed as a way to integrate trauma-informed concepts (i.e., educational content) in a trauma-informed manner (i.e., educational context).^14^ For example, TIME advocates introducing topics that some students might find distressing, such as intimate partner violence (IPV) or taking a sexual history (i.e., educational content), in a learning environment that applies the principles of TIC (i.e., educational context), such as offering peer support for students after a session with distressing material. TIME aims to increase the trauma competency of medical school graduates and improve learner engagement through trauma-informed learning environments.^14^

In the curriculum at Harvard Medical School (HMS), medical and dental students interview patients as early as their first month of school and begin practicing physical examination the following month.^15^ Given the ubiquity of trauma, students encounter traumatic disclosures early on and need skills to respond to them. For this reason, an HMS student-faculty interest group advocated for the inclusion of TIC in multiple areas of the HMS curriculum. Members of the group worked with faculty from the interviewing and communications skills component of the first-year clinical skills course to design a new learning session to provide first-year medical and dental students with explicit instruction in trauma-informed communication skills during the medical interview and a framework to screen for and respond to disclosures of trauma.

A review of *MedEdPORTAL* provided several examples of UME-level education around TIC including trauma-informed approaches to the physical exam,^16^ instruction on adverse childhood experiences,^17^ and trauma-informed approaches to the care of female sexual assault survivors.^18^ Several additional resources have been published recently regarding teaching medical students how to conduct IPV screening, but none that introduced TIC.^19–21^ This is the first session to teach first-year medical and dental students how to screen universally for diverse experiences of trauma and how to respond to disclosures of trauma in a trauma-informed manner.

## Methods

We developed a 3.5-hour session to introduce TIC communication skills to all 164 first-year medical and dental students who matriculated during the 2018–2019 school year. Our group consisted of students and faculty at HMS in collaboration with multidisciplinary faculty at Brigham and Women's Hospital (BWH), including nurse practitioners and social workers. We presented the session within the interview and communication skills component of the first-year clinical skills course in January 2019. The session included three 1-hour segments: a live interview demonstration with a patient, a discussion session in small groups, and case-based role-plays. The session's design and implementation followed TIME principles of content and context: We codesigned all aspects of the session in collaboration with senior students and multidisciplinary clinicians with expertise in TIC; we empowered senior students to take on a variety of leadership roles; and we incorporated peer supports throughout the session.^14^

Groups of two or three student-facilitators and faculty-facilitators coled the small-group discussion and role-play sessions as described below. We assumed neither the student-facilitators nor faculty-facilitators had expertise in TIC. Before the session, we provided all facilitators with a guide (Appendix A) that suggested strategies for leading the small-group sessions in a trauma-informed manner, discussion questions based on the demonstration videos, and instructions for the role-plays. We invited all facilitators to a 1-hour training, which provided the opportunity to view video demonstrations of patient interviews and discuss these and other teaching materials and methods. During the training, attendees also took part in trauma-informed facilitation to learn and practice cofacilitating the session with their student-facilitators.

We designed the TIC session to be interactive, with minimal time spent in lecture and maximal time spent in small-group discussion, hands-on application, and reflection. This session required students to complete preparatory work, consistent with the case-based collaborative learning (flipped classroom) model used in the HMS curriculum.^15,22^ The preparatory assignment included a series of didactic concept videos and simulated patient interview videos (Appendices B, C, and D) totaling 75 minutes. We supplied students with a student guide (Appendix E) to provide anticipatory guidance for what to expect during the session. Because exposure to the course material on trauma might be distressing to some students, we provided a content advisory and recommendations on how to move through the preparatory work. Suggestions included viewing preparatory material with a plan to access support resources afterward if needed; watching the preparatory material with trusted classmates; and taking frequent breaks while working through the material.

The first concept video defined trauma, identified its health effects, and introduced the theory of TIC (Appendix B). The second concept video instructed students on how to use open-ended questions to screen for trauma, how to apply the principles of TIC to IPV screening, and how to respond appropriately to disclosures of trauma (Appendix C). The last set of videos simulated patient encounters, demonstrating how each of the six TIC principles can be incorporated into clinical practice (Appendix D). Each video demonstrated a non-trauma-informed interview followed by a trauma-informed interview and ended with a set of reflection questions. Multidisciplinary members of the BWH TIC Education Committee designed the video demonstrations in collaboration with our group and in partnership with HMS Media Services.

We structured the session as follows:

1. Large-group session (60 minutes)
  ○ Introduction and didactic review (10 minutes)
  ○ Live interview demonstration (50 minutes)
2. Break (15 minutes)
3. Small-group discussion and reflection session (60 minutes)
4. Break (15 minutes)
5. Small-group role-plays (50 minutes)
  ○ Student A as provider, Student B as patient (15 minutes)
  ○ Reflection (10 minutes)
  ○ Student B as provider, Student A as patient (15 minutes)
  ○ Reflection (10 minutes)
6. Wrap-up and postsession survey (10 minutes)
7. Student-only healing space (optional, 60 minutes)

The first hour began in a lecture hall with a 10-minute didactic review of TIC principles. Students then observed a clinician with expertise in TIC inquire about trauma, respond to the patient's disclosure, apply universal trauma education, and provide further counseling in a live interview demonstration. Following the observed interview, students had an opportunity to ask questions of the patient and/or clinician.

The patient interviewed was known to one of our faculty and had previously expressed an interest in teaching medical students about trauma. To address the educational context and prepare for the session, the clinician interviewer and patient met twice beforehand, once by phone and once in person. In these meetings, our clinician interviewer obtained informed consent, reviewed what to expect during the session, previewed the questions that would be asked, and established strategies to use if the patient became distressed (i.e., a signal to slow down or stop the interview). The patient's primary care physician was also in the audience within the patient's line of sight for additional support. Following the session, one of our student-facilitators made themselves available to debrief with the patient.

Following the large-group session, there was a 15-minute break, in which we encouraged students to seek guidance or additional support, as needed.

Following this break, students moved into small groups. Small groups comprised six to eight students along with one to three faculty. These small groups had been working together for 5 months in the interview and communication skills course. Along with their usual faculty educators, most groups had additional facilitation from a TIC expert from a multidisciplinary pool recruited from four HMS-affiliated hospitals, as well as a third- or fourth-year medical student-facilitator. Student-facilitators led discussions and served as a peer support throughout the session. In the end, each group had at least one faculty-facilitator and one student-facilitator for the small-group discussions.

To promote safety and attend to the educational context, facilitators began the reflection session by outlining expectations: confidentiality, assuming positive intent while attending to impact, and respect (no/limited technology). Next, facilitators encouraged students to share any reflections on the preparatory assignment or the live interview demonstration. Finally, facilitators asked students to identify how the provider in the live interview demonstration highlighted the principles of TIC. Groups discussed the assigned reflection questions, and in the remaining time, they created an agenda of topics and reflection questions associated with the video demonstrations for discussion.

Following a second 15-minute break, students spent the last hour of the session in the same small groups with the same facilitators. Students participated in two case-based role-plays (Appendix F) designed to allow them to practice a trauma-informed interview and respond to a trauma disclosure. They were also given a one-page conversation guide (Appendix G), which summarized sample language to use when screening for trauma and responding to trauma disclosures, to guide role-plays. Prior to this session, students had several experiences with role-play simulation during the first-year clinical skills course. After each role-play, facilitators asked students to spend 10 minutes reflecting together on the cases. We created these reflection exercises to help students assess their emotional reactions to trauma disclosure in a safe and supportive educational context. The students switched roles and repeated the exercise with a different case.

In a final effort to attend to the educational context and offer peer support, the medical students in our working group organized an optional, confidential, student-only healing space for first-year students immediately following the session. The healing space lasted for 1 hour and was facilitated by third- and fourth-year medical students with a background in TIC. It was open-ended, and student-facilitators invited first-year students to share reflections and reactions from the session.

To assess the session's effectiveness, students completed pre- and postsession surveys. We embedded the surveys into the session's online course page. The presession survey asked students to assess their knowledge of TIC and their self-reported comfort with screening for and responding to disclosures of trauma, and to identify if they had received a disclosure of trauma before the session (Appendix H). The postsession survey asked students to reassess their knowledge of TIC, report their comfort with screening for trauma and responding to disclosures of trauma, and to offer feedback on the session itself. Five months later, the same group of 164 first-year medical and dental students participated in another TIC educational session, at which time we asked them to complete a follow-up survey. The follow-up survey asked students to identify if they had received a disclosure of trauma following the session, to assess their comfort with screening for and responding to disclosures of trauma, and to identify practice changes following the session. The TIC communication performance assessment (Appendix I) can also be used to assess the skills of the learners.

We used Qualtrics software (Core XM) to collect presession, postsession, and follow-up survey data. We linked data through anonymous survey identifiers. We used 100-point scales for questions regarding comfort with screening for and responding to disclosures (0 = *not at all comfortable*, 100 = *completely comfortable*). We calculated means and standard deviations for students’ self-reported comfort scores and compared them by a two-tailed paired Student *t*-test analysis (α = .05). We analyzed free text for thematic content. Coauthor Taylor Brown manually read through all survey responses. For questions asking about the strengths and areas of improvement, Taylor Brown recorded how many responses commented on specific aspects of the session. For free-text items related to trauma disclosure, Taylor Brown recorded the types of trauma using prespecified categories: childhood abuse; sexual violence; physical violence, violence, or abuse toward a loved one; and structural violence. The postsession survey used a 5-point Likert scale to determine satisfaction with the session and effectiveness of the session at meeting learning objectives (1 = *not at all effective*, 5 = *extremely effective*). We collected 5-month follow-up data on self-reported comfort and practice changes. The data collected from these surveys were part of educational quality improvement and therefore qualified for an institutional review board waiver based on institutional standards at HMS.

## Results

Out of 164 participating students, 124 paired responses were recorded (76% response rate). For the 5-month follow-up data, 82 students completed the survey (50% response rate).

Before the session, 34% of students reported having received at least one disclosure of trauma from a patient within the first 5 months of medical school. Types of disclosure included the following: sexual violence (29%); physical violence and abuse (25%); childhood sexual abuse (24%); and loss of a loved one (14%). Rated on a 100-point scale (0 = *not at all comfortable*, 100 = *completely comfortable*), students’ self-reported baseline mean comfort levels with screening for trauma, responding to trauma disclosure, and counseling patients with positive IPV screens were 30 points (*SD* = 24), 35 points (*SD* = 25), and 25 points (*SD* = 22), respectively.

After the intervention, students’ mean comfort level with trauma screening increased to 56 points (*SD* = 20, *p* < .001). Students’ mean comfort level with responding to disclosures increased to 55 points (*SD* = 21, *p* < .001), and their mean comfort level with counseling patients who screened positive for IPV increased to 50 points (*SD* = 23, *p* < .001). Students expressed high overall satisfaction with the session, with 87% of students reporting that the session was *very effective* or *extremely effective* at meeting the educational objectives. When asked about the most effective session elements, students identified in their free-text responses the live interview demonstration (40% of all respondents), the case-based role-play exercises (29%), the video demonstrations in the preparatory materials (8%), and the role of student and faculty facilitators (5%).

The results of the follow-up survey administered 5 months after the intervention indicated that the observed increase in comfort with trauma screening persisted (*M* = 57, *SD* = 19, *p* = .55), as did the observed increase in comfort with responding to disclosures (*M* = 58, *SD* = 19, *p* = .65). The number of students who received at least one disclosure of trauma nearly doubled to 64% at 5 months postintervention (10 months after matriculation) compared to 34% when the initial TIC session took place (5 months after matriculation). Of responding students, 73% reported changes in their clinical practices following the session. These changes included the use of standard language for trauma screening (30% of survey responses), including a ubiquity statement before discussing trauma (e.g., “I ask this of all my patients since trauma is so common”; 7% of survey responses), and minimizing the details shared about a trauma (e.g., “We don't have to go into all of the details”; 3% of survey responses), also known as containment.

## Discussion

We developed an introductory program on TIC communication skills for first-year medical and dental students to address a critical gap in UME. If a clinician lacks trauma-informed communication skills, patients may be less likely to disclose past or current experiences of trauma and may be at increased risk of being retraumatized during clinical encounters. In addition, clinicians who lack trauma-informed communication skills may be less likely to inquire about trauma, may be more distressed by a patient's disclosure, and may not know how to respond or how to process what the patient has told them. Clinicians may discover that their own trauma responses are provoked by these experiences. As medical schools introduce clinical experiences earlier in the curriculum, it is becoming necessary to integrate trauma-informed communication skills for the safety of both patients and learners. The content of this session provides students with the skills to discuss trauma in a way that minimizes retraumatization and emphasizes strength and resilience.

Our TIC curriculum and educational session represent a feasible educational intervention that participants reported was satisfying and effective. Our results suggest an increase in students’ comfort with TIC communication skills that persists for at least 5 months after the initial educational session. Furthermore, our findings suggest that all medical students can and should learn about trauma and trauma-informed communication skills early in their education, optimally as soon as they begin to interact with patients. This is crucial, as we found that over one-third (34%) of students received at least one trauma disclosure before receiving training on how to respond to such disclosures, and that 10 months after matriculation, the percentage of students that received at least one trauma disclosure nearly doubled to 64%.

The development of this session relied heavily on student advocacy, which remained one of the session's greatest strengths. Through coproduction of course material with senior students and multidisciplinary TIC experts, we created a robust library of TIC curricular content. We recommend that all institutions seeking to develop a similar session coproduce it with students and multidisciplinary clinicians. Additionally, our session relied heavily on the HMS curricular structure in both recruiting faculty facilitators and creating cohesive small groups. For our intervention to be successfully implemented at other medical schools, educators would need to identify similarly cohesive small groups within their own curricular structure. Other institutions may also find it challenging to recruit and train the number of facilitators required for small-group sessions.

An important consideration in teaching TIC content is how to minimize distress for students. We know that teaching trauma content without a trauma-informed approach is potentially traumatic for students and faculty.^14,23,24^ Embodying or pretending to be a patient with lived experience of trauma can be distressing for students, particularly for students with their own experiences with adversity. Ideally, students should practice responding to disclosures with professional standardized patients. The next best alternative would be faculty and near-peer senior medical students assuming the patient role during practice role-plays. If neither of the aforementioned options are feasible, we encourage facilitators to empower small groups to choose the role-play methods that work best for their learners. One example of this would be to allow students to self-select into groups in which at least one student is comfortable in the patient role. Facilitators must be careful to normalize opting out of the patient role. Whatever the arrangement, students should be encouraged to verbally practice responding to disclosures. Finally, we have updated the role-plays to include standardized patient instructions. This will help to standardize the exchanges so that different students have similar learning experiences, as well as preventing participants from having to imagine how to disclose trauma.

This session served as an introduction to TIC not only for first-year students but also for many of our teaching faculty. Faculty development was key to the success of the initial session and will be key to the integration of TIC curricular content and context moving forward. As a result, we are currently designing a standardized faculty development curriculum in the format of asynchronous online modules to support our faculty in teaching TIC clinical skills. In the future, we hope to share these faculty development modules to assist with TIC content integration across other institutions.

One limitation of the session is that the field has moved away from trauma screening and now embraces trauma inquiry.^25^ One main difference between screening and inquiry is that when pursuing trauma inquiry, the goal is not a disclosure of trauma. Trauma inquiry focuses on the symptoms and impacts of trauma experiences on one's health and well-being and seeks to prevent the retraumatization that may accompany the act of recounting a traumatic experience. Inquiry follows a tiered approach that allows patients to control whether and how much to disclose and only advises continued questioning when an immediate safety risk is present. Our original session used a similar open-ended question approach, but still called the technique screening. Therefore, the term *screening* is reflected in the teaching material and our survey results. Using the materials provided, instructors can nevertheless teach trauma inquiry. In subsequent iterations of the session, we have emphasized that disclosure is not the goal of trauma inquiry and have recommended screening only as a means of safety assessment, such as the case of ongoing IPV. Students are instructed to be mindful of how many times a patient must retell their story, avoid asking for details if not treating acutely, and encourage patients to disclose only as much as they feel comfortable sharing. As students’ skills progress, they can begin to explore the effects of trauma on a patient's health.

Another limitation of our study is that student assessment relied on self-reported impressions without applying objective measures of learning, such as knowledge questions. Additionally, the survey items did not allow us to assess our stated learning objectives. Finally, for the questions asking students to report on their comfort with TIC clinical skills, we do not know the effect of using a 100-point scale as opposed to a more standard 5-point Likert scale. In future iterations of the session, we plan to use knowledge-based questions to assess each stated learning objective and convert 100-point scales to more standard 5-point Likert scales. We developed a TIC communication performance assessment (Appendix I) to assist other institutions in assessing students’ clinical skills. Development of robust assessments for TIC clinical skills is an active area of research for our group. We have developed TIC competencies to guide student instruction, are crafting Entrustable Professional Activities to track skills development during clerkships, and are developing OSCEs to assess clinical skills acquisition. These projects are ongoing at the time of publication. Future studies may include focus groups with students’ patients to understand the patient experience when students utilize a TIC approach to care.

To our knowledge, this publication is the first to describe a session introducing first-year medical and dental students to a trauma-informed approach to obtaining the patient history, screening for and inquiring about trauma, and responding to trauma disclosures. This session represents one of several efforts at our institution to increase students’ understanding of the definition and health effects of trauma and the importance of adopting TIC as a universal approach to patient care.^14^ We hope this work represents a step toward the creation of a trauma-informed educational context via trauma-informed session facilitation and explicit teaching on self-care and peer-support strategies for students facing the sequelae of trauma in their patients, their colleagues, and themselves.

## Appendices

Facilitator Guide.docxTIC Introduction.mp4TIC Intimate Partner Violence and Screening.mp4Video Demonstrations.mp4Student Guide.docxTrauma-Informed Care Role-Play Cases.docxConversation Guide.docxPre-, Post-, and Follow-Up Surveys.docxTIC Communication Performance Assessment.docx
*All appendices are peer reviewed as integral parts of the Original Publication.*
