# Supplementary material for: A Trauma-Informed Approach to the Medical History: Teaching Trauma-Informed Communication Skills to First-Year Medical and Dental Students
Source: MedEdPORTAL. 2021 Jun 7;17:11160. doi: 10.15766/mep_2374-8265.11160 (PMC8180538; doi:10.15766/mep_2374-8265.11160)
Supplement: Supplementary file 1 — Facilitator Guide.docxTIC Introduction.mp4TIC Intimate Partner Violence and Screening.mp4Video Demonstrations.mp4Student Guide.docxTrauma-Informed Care Role-Play Cases.docxConversation Guide.docxPre-, Post-, and Follow-Up Surveys.docxTIC Communication Performance Assessment.docx [file mep_2374-8265.11160-s001.zip › A. Facilitator Guide.docx]

**An Introduction to Trauma-informed Care**

Faculty Guide

Prepared by Pooja Mehta, HMS ‘19

**Background:**

In this session, we will be introducing trauma-informed care (TIC). A trauma-informed approach to patient care recognizes that many of our patients have experienced or witnessed traumatic events in their lives and that these experiences can have a significant impact on their health. As providers, we aim to recognize trauma and its impact on health, address it with our patients, and avoid re-traumatization. TIC is an approach to patient care rather than a prescribed set of practices.

In the preparatory work and in this session, we will explicitly be discussing trauma. This topic can bring up a number of emotions and difficult reactions in all of us. If at any point you feel distressed, uncomfortable, or unwell, please practice self-care in whatever way is most helpful for you. Depending on where you are at emotionally or otherwise, **consider doing this preparatory work around colleagues, friends, or other support structures.** You may also want to consider scheduling some time for self-care after completing the preparatory work and after the session.

**Educational Objectives**

By the end of this activity, learners will be able to do the following:

1. Define trauma and explain its prevalence and health impacts;
2. Describe the six principles of trauma-informed care (TIC) as defined by the Substance Abuse and Mental Health Services Administration (SAMHSA);
3. Demonstrate a trauma-informed approach to the patient history;
4. Screen for and inquire about trauma, including intimate partner violence, using open-ended questions or a validated screening tool and respond appropriately to disclosures of trauma using TIC principles.

**Preparatory Work: ~75 minutes**

*Please note: the videos assigned for you are identical to the student preparatory work*

**Please watch videos in the order listed:**

- Introduction to TIC Concept Video (13 minutes)
- TIC Applied to Intimate Partner Violence Concept Video (17 minutes)
- TIC Video Demonstrations Concept Video (35 minutes)
  - TIC Video Demonstration - Introduction
  - TIC Video Demonstration - Safety
  - TIC Video Demonstration - Trust and Transparency
  - TIC Video Demonstration - Peer Support
  - TIC Video Demonstration - Culture, Historic, Gender Issues
  - TIC Video Demonstration - Collaboration and Mutuality
  - TIC Video Demonstration - Empowerment, Voice, and Choice
- Read faculty guide

**Session Agenda**

- Patient Clinic* (1 hour)
- Break (15 minutes)
- Small group debriefing and reflection session (1 hour)
- Break (15 minutes)
- Small group role plays (50 minutes)
  - Student A as provider, Student B as patient (15 minutes)
  - Reflection (10 minutes)
  - Student B as provider, Student A as patient (15 minutes)
  - Reflection (10 minutes)
- Wrap-up (10 minutes)
- **Students-only** Healing Space (optional, 1 hour)

*Given the nature of this topic, it is in our patient’s best interest to have choice in whether they attend the session on the day of the session. If our patient is unable to attend the session, we will have a multidisciplinary panel of providers with expertise in TIC.

**Small Group Breakouts: Debriefing and Reflection Session (1 hour)**

**Facilitating Using the Principles of Trauma-informed Care**

Facilitators will in most cases be working with 6-8 first year students. Assignments will be sent to all facilitators in advance of the session. Facilitators are a multidisciplinary group including faculty from different specialties who teach in the course, clinicians with expertise in TIC, and students who are interested and experienced with TIC. Facilitators may be working alone or in a pair. For facilitator pairs that include a student-facilitator, we hope that the student-facilitator will take the lead in the debriefing and reflection session. The student’s co-facilitator should hopefully serve in a supporting role, offering expertise and suggestions when helpful. Facilitator pairs should try to take a moment after the afternoon session is completed to debrief about what went well and what changes they might make in the future.

The way that we, health professionals, communicate with each other and with student learners should be trauma-informed. You can demonstrate these principles in the way that you facilitate the discussion.

To promote **safety**, please begin by reinforcing the rules of the space: respect (no/limited technology), confidentiality, and assume positive intent while attending to impact.

Next, if you are new to the group, please introduce yourself and share what brought you to TIC and why you wanted to facilitate this session. If you are known to the group, please share a brief reflection about what it has been like reviewing this material at your current level of training/practice. This can help to create a safe space between you, who is in the position of power, and the students. This promotes **trust and transparency**.

To promote **peer support** and **empowerment, voice, and choice**, ask each student to reflect on the prep work and the previous session (patient clinic or provider panel). Empower students to share as much or as little as they wish. If they want to pass, make that okay. This gives students the opportunity to share any emotional responses they had and for the group to support them. This can take as long as the students desire and is the most important part of this debrief. Before getting into a case or discussion questions, allow everyone the opportunity to reflect. Be mindful of **culture, historic, and gender issues** as the students share their reflections. In addition, as students reflect, your role is to listen and comment with reflective, empathetic statements.

Finally, to promote **collaboration and mutuality**, set the agenda for the rest of this hour together. The suggested activity is to discuss observations and questions that students have about the topics of TIC and intimate partner violence, including the concept videos viewed in advance of the session. The hope is that there will be time to discuss one or more of the video demonstrations. Allow the students as a group to decide which cases presented in the video demonstrations they would like to review or discuss further. Take the remaining time to discuss those cases. The one-liners and reflection questions are listed on the next page.

**Please set a timer so that you end promptly after 1 hour to take a 15-minute break. The break gives students the opportunity to take a few moments for themselves, seek peer support, and ask questions privately and is as important as the group sessions.**

Concept Video Reflection Questions

Safety

Zara (she/her/hers) is a 44-year-old, white, cisgender, female patient who is 8 weeks pregnant coming in for an initial prenatal appointment.

1) In this scenario, the provider screens for intimate partner violence and offers to connect the patient to a program called Passageways. How is the provider’s wording and demeanor different in each version?

2) How does the patient respond to the ways the provider relays his concern for her? How might you express concern for your patient’s well-being in trauma-informed ways?

3) What is the relationship between body positioning and power dynamics? How might physical stance be of particular importance to a patient experiencing loss of power in their personal relationship?

*Potential Responses*

1. *In the first version, the provider is standing above the patient and introduces the inquiry as an something “he forgot,” which may diminish the value of the inquiry. In the trauma-informed version, the provider is sitting below the patient and he introduces the idea of universal trauma inquiry prior to proceeding with his questions. A more subtle difference in the second version is the provider uses a softer, less severe tone of voice. This helps to put the patient at ease as she discusses a challenging topic.*
2. *In the first version, the provider often says “okay good,” when the patient makes comments like “it’s not that bad,” which may diminish the significance of her life experienes. In the trauma-informed version, the provider repeats back what he hears to the patient. By demonstrating that he is listening, the patient can feel more secure in the relationship by feeling heard.*
3. *Standing above the patient imposes a sense of power over the patient. When a patient has lost power in other relationships, it can be re-traumatizing to experience that loss of power in therapeutic relationships. This impairs the provider’s ability to connect with the patient in a therapeutic relationship.*

**Key teaching points**

1. **Body positioning (at or below eye level of the patient) and keeping adequate physical distance are important nonverbal cues to establish psychological safety.**
2. **Responding to patient emotions with empathy will help improve their psychological safety.**

Trustworthiness and Transparency

Jean (she/her/hers) is a white, cis-gender, female patient in her late 30’s and is seeing her doctor for her annual physical. She has a history of insomnia and no other major medical issues.

1) In the TIC version, the provider states, “we review these questions with everyone.” Is this statement effective? Why or why not?

2) The provider used a written safety assessment instead of verbally asking questions. Why might they have done so? Which method do you think is better?

3) The provider uses the question, “is there anything you want to add?” Is this a question you would feel comfortable incorporating?

*Potential Responses*

1. *Normalizing statements can reduce any judgement patients might feel given the personal and sensitive nature of the questions.*
2. *Some patients find it easier to consider and answer questions about trauma through nonverbal, written communication. Other patients may respond better to a conversation. There is no clear answer and both methods can be utilized.*
3. *Yes, in this context an open-ended question invites the patient to participate in the way they feel is best.*

**Key teaching points**

1. **Setting an agenda for the visit and providing anticipatory guidance are ways to add transparency and help to build trust between provider and patient.**

Peer Support

Two first-year medical students are shown talking after a day in clinic.

1) Why do you think the student in the TIC scenario said, “I wonder: is it just the two of us?” Why is this important?

2) In the TIC version, one student validates the other student in saying, “It makes me feel less alone in this”. Can you imagine being open to your peers in this way? Why or why not? What would you need to be able to be comfortable saying something like this?

3) How do you feel, hearing the student say they ‘don’t want to pretend it’s not happening?’ How do you think this relates to the TIC model?

*Potential Responses*

1. *At times, providers or patients may feel that what they are experiencing is abnormal when in fact, many people share their feelings. Feelings of isolation can increase burnout and negatively affect well-being.*
2. *Some students may express concerens about trust, vulnerability, fear, excitement, etc. Some may say they need to have a personal or trusted relationship. Others may say they need to be asked and so may try to ask their peers to share.*
3. *Some students may express that peers sometimes pretend disclosures of trauma do not happen or that those disclosures do not affect them. TIC aims to recognize that these conversations happen regularly and impact everyone (students, patients and providers).*

**Key teaching points**

1. **Peer support is a valuable tool to improve individual and collective well-being.**

Cultural, Historic, and Gender Issues

Romelia (she/her/hers) is 38-year-old, Latina cisgender, female patient who presents to the Emergency Department with a fever, headache, stiff neck, and blurred vision.

1) Discuss the power dynamics in the two versions of this scenario. What were the key differences? What statements created a power dynamic and which statements empowered the patient? Consider tone, language, and body positioning for each question.

2) What are takeaways that you would use when meeting an acutely ill patient for the first time? What might be difficult to incorporate?

*Potential Responses*

1. *In the first version, the provider is standing above the patient and does not clearly introduce themselves or their role on the team. In the second, the provider is sitting, formally introduces themself and their role, and asks the patient how they would like to be addressed. The provider also uses a softer, even paced tone.*
2. *Since acutely ill patients require a more rapid evaluation than other patients, it may be challenging to incorporate TIC skills as a beginner. Often a patient’s acute medical needs may prevent the team from being able to offer complete explanations. However, we always have time to clearly introduce ourselves and our role on the care team, as well as confirm how the patient would like to be addressed. This helps establish trust and safety even in medical emergencies.*

**Key teaching points**

1. **Providers should always confirm with the patient how they would like to be addressed and identify themselves and their role on the team.**
2. **Providers should consider the influence cultural, historic, and gender-based experiences have on patients’ interactions with the healthcare system.**

Collaboration and Mutuality

Carol (she/her/hers) is a middle-aged, white, cisgender, female patient admitted to the hospital for chronic kidney failure.

1) Describe the power differential in the two versions of the scenario. What phrases assist the provider in creating partnership and sharing power?

2) The patient describes loss of agency and pressure when it comes to decision-making. In what ways does the medical system perpetuate these dynamics?

3) When the patient discloses the dynamics in her marriage, how does the provider create an opportunity to build trust with the patient in the TIC version? How might this enhance her sense of safety?

*Potential Responses*

1. *By recognizing the patient’s concerns about her PCP and asking, “It sounds like the primary care doctor is not the right person?” the patient was able to share that she found a nurse practitioner that she feels more comfortable following up with. Then together, patient and provider were able to come up with an outpatient follow-up plan the patient was comfortable with and therefore more likely to embrace.*
2. *The medical system requires patients to constantly make decisions about their health, often with little time and incomplete understanding. These dynamics recapitulate other life experiences in which patients feel a loss of control, much like how this patient describes the agency taken from her by her husband.*
3. *The provider states, “it sounds like that’s been really hard – would you be willing to talk to a social worker about some of these dynamics so that we can come up with a plan you feel comfortable with?” When the provider responds to the patient’s emotions first, the patient is more likely to feel safe.*

**Key teaching points**

1. **To promote collaboration and mutuality, providers can develop recommendations with patient input instead of presenting a plan to the patient, then immediately directing them to follow that plan.**

Empowerment, Voice, and Choice

Maria (she/her/hers) is a 35-year-old, Latina, cisgender, female patient with a history of trauma, recently diagnosed with breast cancer, preparing for mastectomy with no reconstruction.

1) How ​did the provider allow or not allow the patient to feel heard and understood in each scenario?

2) How did the TIC scenario address the TIC principle of patient empowerment? What was the medical student able to convey to the patient in each scenario? How might these messages help someone like this patient, who has a history of trauma and is now faced with a new health crisis?

*Potential Responses*

1. *In the first version, the provider made statements like, “Don’t worry”, which, while intended to soothe the patient’s anxiety, may instead have invalidated the patient’s feelings. In the second video, the provider validated the patient’s feelings and empowered her to verbalize her concerns.*
2. *The provider validated the patient when she raised her concerns and empowered her to verbalize her concerns prior to her surgery. Trauma is often directly associated with disempowerment and loss of control over a situation. A new health crisis can be triggering and re-traumatizing.*

**Key teaching points**

1. **Empowerment, voice, and choice can be utilized in every patient encounter.**
2. **Patients with trauma histories can often be triggered by new health crises. We can mitigate these effects by empowering patients, listening to their voices and offering choices.**

**Small Group Breakouts: Role Play (50 minutes)**

Students will practice two role plays in pairs (see next page). Student A will be the provider for the first case, patient for the second and vice versa for Student B.

Please explain that they will be doing two role plays to practice having trauma-informed conversations and highlight that they are encouraged to use the conversation guide to assist them in asking the questions. The goal of this exercise is not to memorize the phrases, but to practice saying them aloud. In addition, please share the performance assessment checklist with students, so there is transparency about what the specfic goals of the exercise are.

Normalize the discomfort that is often associated with role plays and highlight that this space is meant to be a safe space in which to practice and fumble before asking these questions to real patients.

Embodying or pretending to be a patient with lived experience of trauma can be distressing for students, particularly for students with their own experiences with adversity. It is critical that students be offered a choice about playing the role of a patient with trauma, optimally with an "opt-in" rather than an "opt-out" process to minimize the risk of traumatizing students.

**Please set a timer to keep the group on time.**

Recommended Time Breakdown:

Set the agenda and orient the students to this hour of the session (2 minutes)

Case Scenario #1 (25 minutes)

- Read the case & role play (15 min)
- Read the other perspective and debrief as a group (10 min)

Case Scenario #2 (23 minutes)

- Read the case & role play (13 min)
- Read the other perspective and debrief as a group (10 min)

Wrap-up (10 minutes)

- Please allow students time to complete the post-session survey.

**Optional Reading and Additional Resources:**

- Machtinger EL, Cuca YP, Khanna N, Rose CD, Kimberg LS. From treatment to
  healing: the promise of trauma-informed primary care. Women’s Health Issues. 2015
  May-Jun;25(3):193-7.
- Nadine Burke-Harris TED talk: How childhood trauma affects health across a lifetime
- Raja S, Hasnain M, Hoersch M, Gove-Yin S, Rajagopalan C. Trauma informed care
  in medicine: current knowledge and future research directions. Fam Community
  Health. 2015 Jul-Sep;38(3):216-26.

**Acknowledgments:**

This session was established following advocacy by members of the Harvard Medical School Trauma Informed Care Working Group (TICWG). This session was designed by a multidisciplinary team of students, social workers, nurses, and physicians who have learned immensely from the patients they have cared for.

*Students & Alumni*

- Pooja Mehta, MD, HMS ‘19
- Sarah Berman, MD, HMS ‘20
- Taylor Brown, HMS’ 21
- Katherine McDaniel, HMS ‘21
- Katie Radford, HMS ‘21

*Brigham and Women’s Hospital Trauma Informed Care Working Group*

- Samara Grossman, LICSW
- Annie Lewis-O’Connor, NP, PhD

*HMS Faculty*

- David Hirsh, MD
- David Krieger, MD
- Beverly Woo, MD
