# Supplementary material for: A Trauma-Informed Approach to the Medical History: Teaching Trauma-Informed Communication Skills to First-Year Medical and Dental Students
Source: MedEdPORTAL. 2021 Jun 7;17:11160. doi: 10.15766/mep_2374-8265.11160 (PMC8180538; doi:10.15766/mep_2374-8265.11160)
Supplement: Supplementary file 1 — Facilitator Guide.docxTIC Introduction.mp4TIC Intimate Partner Violence and Screening.mp4Video Demonstrations.mp4Student Guide.docxTrauma-Informed Care Role-Play Cases.docxConversation Guide.docxPre-, Post-, and Follow-Up Surveys.docxTIC Communication Performance Assessment.docx [file mep_2374-8265.11160-s001.zip › E. Student Guide.docx]

**An Introduction to Trauma-informed Care**

Session Guide

Prepared by Pooja Mehta, HMS ‘19

**Background:**

In this session, we will be introducing trauma-informed care (TIC). A trauma-informed approach to patient care recognizes that many of our patients have experienced or witnessed traumatic events in their lives and that these experiences can have a significant impact on their health. As providers, we aim to recognize trauma and its impact on health, address it with our patients, and avoid re-traumatization. TIC is an approach to patient care rather than a prescribed set of practices.

In the preparatory work and in this session, we will be explicitly discussing trauma. This topic can bring up a number of emotions and difficult reactions in all of us. If at any point you feel distressed, uncomfortable, or unwell, please practice self-care in whatever way is most helpful for you. Depending on where you are at emotionally or otherwise, **consider doing this preparatory work around friends, classmates, or other support structures.** You may also want to consider scheduling some time for self-care after completing the preparatory work and after the session. There will be an optional student-only healing space directly following the session.

**Educational Objectives:**

By the end of this activity, learners will be able to do the following:

1. Define trauma and explain its prevalence and health impacts;
2. Describe the six principles of trauma-informed care (TIC) as defined by the Substance Abuse and Mental Health Services Administration (SAMHSA);
3. Demonstrate a trauma-informed approach to the patient history;
4. Screen for and inquire about trauma, including intimate partner violence, using open-ended questions or a validated screening tool and respond appropriately to disclosures of trauma using TIC principles.

**Session Agenda**

- Patient Clinic* (1 hour)
- Break (15 minutes)
- Small group debriefing and reflection session (1 hour)
- Break (15 minutes)
- Small group role plays (50 minutes)
- Wrap-up (10 minutes)
- Students-only Healing Space (optional, 1 hour)

*Given the nature of this topic, it is in our patient’s best interest to have choice in whether they attend the session on the day of the session. If our patient is unable to attend the session, we will have a multidisciplinary panel of providers with expertise in TIC.

**Preparatory Work: ~75 minutes**

**Please complete in the order listed:**

- Introduction to TIC Concept Video (13 minutes)
- TIC Applied to Intimate Partner Violence Concept Video (17 minutes)
- TIC Video Demonstrations Concept Video (35 minutes)
  - TIC Video Demonstration - Introduction
  - TIC Video Demonstration - Safety
  - TIC Video Demonstration - Trust and Transparency
  - TIC Video Demonstration - Peer Support
  - TIC Video Demonstration - Culture, Historic, Gender Issues
  - TIC Video Demonstration - Collaboration and Mutuality
  - TIC Video Demonstration - Empowerment, Voice, and Choice

**Resources**

- Conversation Guide
- Role-Play Cases
- TIC Communication Performance Assessment

**Optional Reading:**

- Machtinger EL, Cuca YP, Khanna N, Rose CD, Kimberg LS. From treatment to
  healing: the promise of trauma-informed primary care. Women’s Health Issues. 2015
  May-Jun;25(3):193-7.
- Nadine Burke-Harris TED talk: How childhood trauma affects health across a lifetime
- Raja S, Hasnain M, Hoersch M, Gove-Yin S, Rajagopalan C. Trauma-informed care
  in medicine: current knowledge and future research directions. Fam Community
  Health. 2015 Jul-Sep;38(3):216-26.

**Acknowledgments:**

This session was established following advocacy by members of the Harvard Medical School Trauma-informed Care Working Group (TICWG). This session was designed by a multidisciplinary team of students, social workers, nurses, and physicians who have learned immensely from the patients they have cared for.

*Students & Alumni*

- Pooja Mehta, MD, HMS ‘19
- Sarah Berman, MD, HMS ‘20
- Taylor Brown, HMS’ 21
- Katherine McDaniel, HMS ‘21
- Katie Radford, HMS ‘21

*Brigham and Women’s Hospital Trauma-Informed Care Working Group*

- Samara Grossman, LICSW
- Annie Lewis-O’Connor, NP, PhD

*HMS Faculty*

- David Hirsh, MD
- David Krieger, MD
- Beverly Woo, MD
