# Supplementary material for: A Trauma-Informed Approach to the Medical History: Teaching Trauma-Informed Communication Skills to First-Year Medical and Dental Students
Source: MedEdPORTAL. 2021 Jun 7;17:11160. doi: 10.15766/mep_2374-8265.11160 (PMC8180538; doi:10.15766/mep_2374-8265.11160)
Supplement: Supplementary file 1 — Facilitator Guide.docxTIC Introduction.mp4TIC Intimate Partner Violence and Screening.mp4Video Demonstrations.mp4Student Guide.docxTrauma-Informed Care Role-Play Cases.docxConversation Guide.docxPre-, Post-, and Follow-Up Surveys.docxTIC Communication Performance Assessment.docx [file mep_2374-8265.11160-s001.zip › G. Conversation Guide.docx]

**Introduction to Trauma Informed Care Session**

Conversation Guide

Prepared by Pooja Mehta, HMS ‘19

Screening for Trauma

*Setting the Stage*

- I would like to take a few moments for us to speak together, just the two of us. This is something that I do with all of my patients. Would that be okay with you?

*Normalize*

- I am going to ask you a few questions about trauma, which we know is incredibly prevalent, so that I can provide the best care for you.

*Ask*

- In an effort to be more trauma-informed, I want to ask - have you had any significant or traumatic life experiences that you think it would be helpful for me to know about?

*Clarify*

- Trauma is a very general term for experiences that have a long-lasting, harmful effect on you. Trauma can be physical or emotional abuse, but it could also be facing financial crisis, or experiencing discrimination.

Assessing for IPV

*Setting the Stage*

- I would like to take a few moments for us to speak together, just the two of us. This is something that I do with all of my patients. Would that be okay with you?

*Normalize*

- An important part of my ability to care for you is to ask a few questions about safety in your relationships. This is something I talk about with all of my patients.

*Ask*

- Is there anyone in your life who is controlling or acting threatening toward you?
- Have you ever felt unsafe in your current or previous relationships?

*Clarify*

- For instance, has anyone told you what to do all of the time, made you feel like you don’t have control over your own actions and perhaps made you feel scared in various ways?
- For instance, has anyone harmed you - physically, emotionally, or in other ways?

Responding to Disclosure

Empathic Statement, offer assistance, and counsel

● I am so sorry that is happening (or has happened) to you. I appreciate you telling me your story. I would like to share some options with you. How does that sound? [Counsel, or say] I am not sure how to best do that, and I would like to look into what those options might be.
