# Supplementary material for: A Trauma-Informed Approach to the Medical History: Teaching Trauma-Informed Communication Skills to First-Year Medical and Dental Students
Source: MedEdPORTAL. 2021 Jun 7;17:11160. doi: 10.15766/mep_2374-8265.11160 (PMC8180538; doi:10.15766/mep_2374-8265.11160)
Supplement: Supplementary file 1 — Facilitator Guide.docxTIC Introduction.mp4TIC Intimate Partner Violence and Screening.mp4Video Demonstrations.mp4Student Guide.docxTrauma-Informed Care Role-Play Cases.docxConversation Guide.docxPre-, Post-, and Follow-Up Surveys.docxTIC Communication Performance Assessment.docx [file mep_2374-8265.11160-s001.zip › H. Pre-, Post-, Follow-Up Surveys.docx]

**Pre-Survey**

Before completing any of the preparatory work for this session, please complete this brief survey regarding your knowledge and comfort level with trauma and trauma screening.

Please create a unique six-digit survey ID that will be used to link your pre and post survey. This should be some string of numbers that is memorable to you, but not identifiable, for example parents birthday and a street address you have lived at.

Free Text Response

How would you define trauma?

Free Text Response

Have you ever received a patient disclosure of trauma?

Yes

Maybe

No

If you responded yes to the previous questions, what did your patient disclose?

Free Text Response

How prevalent do you think trauma is among patients?

0-100% sliding scale

How prevalent do you think trauma is among providers?

0-100% sliding scale

How comfortable do you feel screening for a history of trauma?

0-100% sliding scale

How comfortable do you feel responding to patient disclosures?

0-100% sliding scale

How comfortable do you feel counseling a patient if they disclose to you that they have experienced or are experiencing intimate partner violence?

0-100% sliding scale

**Post-Survey**

Now having completed the session on trauma-informed care, please answer the following questions.

Please enter the unique six-digit survey ID that you created for the pre survey.

Free Text Response

How would you define trauma?

Free Text Response

Have you ever received a patient disclosure of trauma?

Yes

Maybe

No

If you responded yes to the previous questions, what did your patient disclose?

Free Text Response

How prevalent do you think trauma is among patients?

0-100% sliding scale

How prevalent do you think trauma is among providers?

0-100% sliding scale

How comfortable do you feel screening for a history of trauma?

0-100% sliding scale

How comfortable do you feel responding to patient disclosures?

0-100% sliding scale

How comfortable do you feel counseling a patient if they disclose to you that they have experienced or are experiencing IPV?

0-100% sliding scale

How effective was the session at meeting the outlined learning objectives? (0-10 with 10 being met all objectives effectively)

Extremely effective/ Very effective/ Moderately effective/ Slightly effective/ Not at all effective

What was particularly effective about the session?

Free Text Response

What would you change for next year?

Free Text Response

**Follow-Up Survey**

Thank you for participating in our follow up survey.

As you recall, in January 2019, you participated in an Introduction to Trauma-informed Care as part of your POM course. Please answer the following questions in thinking about the skills taught at that session.

Have you ever received a patient disclosure of trauma?

Yes

No

If you responded yes to the previous questions, what did your patient disclose?

Free Text Response

How often do you screen for a history of trauma?

Never

Rarely

Sometimes

Often

Always

How comfortable do you feel screening for a history of trauma?

0-100% sliding scale

How comfortable do you feel responding to patient disclosures?

0-100% sliding scale

Did you communicate differently with patients based on the introduction to trauma-informed care session?

Yes/

No

If you responded yes to the previous question, please describe how you changed your practice?

Free Text Response
