# Supplementary material for: A Trauma-Informed Approach to the Medical History: Teaching Trauma-Informed Communication Skills to First-Year Medical and Dental Students
Source: MedEdPORTAL. 2021 Jun 7;17:11160. doi: 10.15766/mep_2374-8265.11160 (PMC8180538; doi:10.15766/mep_2374-8265.11160)
Supplement: Supplementary file 1 — Facilitator Guide.docxTIC Introduction.mp4TIC Intimate Partner Violence and Screening.mp4Video Demonstrations.mp4Student Guide.docxTrauma-Informed Care Role-Play Cases.docxConversation Guide.docxPre-, Post-, and Follow-Up Surveys.docxTIC Communication Performance Assessment.docx [file mep_2374-8265.11160-s001.zip › I. TIC Communication Performance Assessment.docx]

Trauma-informed Care Communication Performance Assessment

The table below is an assessment tool that can be used by role-play observers. For transparency, this tool should be shared with students in advance, so they can be aware of and learn the elements of this assessment. Examples of language students could use are included in the conversation guide.

| **Domain** | **Did** | **Sometimes Did** | **Did Not Do** | **Specific Feedback** |
| --- | --- | --- | --- | --- |
| Positions themself at eye level with open body posture |  |  |  |  |
| Adjusts tone of voice in response to patient |  |  |  |  |
| Listens without interruption |  |  |  |  |
| Provides anticipatory guidance before inquiring about trauma |  |  |  |  |
| Explains that inquiring about trauma is standard practice |  |  |  |  |
| Inquires about trauma |  |  |  |  |
| Provides clarifying statements about the definition of trauma |  |  |  |  |
| Responds to the disclosure of trauma with empathetic statements |  |  |  |  |
| Offers resources to patient |  |  |  |  |
