# Supplementary material for: A Trauma-Informed Approach to the Medical History: Teaching Trauma-Informed Communication Skills to First-Year Medical and Dental Students
Source: MedEdPORTAL. 2021 Jun 7;17:11160. doi: 10.15766/mep_2374-8265.11160 (PMC8180538; doi:10.15766/mep_2374-8265.11160)
Supplement: Supplementary file 1 — Facilitator Guide.docxTIC Introduction.mp4TIC Intimate Partner Violence and Screening.mp4Video Demonstrations.mp4Student Guide.docxTrauma-Informed Care Role-Play Cases.docxConversation Guide.docxPre-, Post-, and Follow-Up Surveys.docxTIC Communication Performance Assessment.docx [file mep_2374-8265.11160-s001.zip › F. Trauma-Informed Care Role-Play Cases.docx]

Case Scenarios

ICS Session on Trauma Informed Care

*Disclaimer: These case scenarios are based on real patient experiences but details have been changed including any personal identifiers.*

**Case 1:** Prepared by Samara Grossman, LICSW

**Provider Perspective:**

You are meeting a patient Ming, 25, who is coming to see you for a new patient visit. They have a history of chronic low back pain and they are noted to have high blood pressure during today’s clinic visit.

Ask about the patient’s past medical history and obtain a social history.

**Patient Perspective:**

Your name is Ming. You are here to establish care with a new provider. You have a past medical history of hypertension and chronic back pain. You are currently not on any medications, because you have not established care with a provider in some time. You have been in a relationship with your partner for about five years. They have been very generous with their money and time and you feel they are kinder to you than anyone else has been in your life. You grew up in a working-class family. Both of your parents suffered from alcohol use disorder. When they drank alcohol, they grew angry and frequently beat you and your siblings. You do not consume alcohol, because your parents suffered from alcohol use disorder. You do not smoke or use any other substances. When you met your partner, they made you feel special and also helped you escape living with your parents. Recently, they proposed to you. They have also physically abused you. You have been struck in the back several times over the past couple of years, which has resulted in your chronic back pain. The pain is dull and aching, 4/10 on the pain scale, and localized to the lower back without radiation. You have no associated neurologic symptoms (no numbness, weakness, tingling, saddle anesthesia, loss of bowel/bladder function). Nothing has made the pain better, though sometimes the pain feels worse. You aren’t sure why your back hurts more at some times and less at other times; there is no clear pattern. In clinic, you also feel stressed because you are worried your doctor will judge you for marrying someone who has hurt you.

The provider’s objective for this conversation is to ask about past medical history and obtain a social history. Of note, this case is written to be gender neutral. You may choose the gender identities of the patient and the patient’s partner and disclose appropriately.

- If the provider asks an open-ended question about when your back pain started, you can say that it started years ago when your partner struck you.
  - If the provider asks follow-up questions about your safety, you may disclose that your partner sometimes hits you, but that mostly they are very kind to you and that you are engaged. Express concern that the provider will judge you for marrying someone that has hurt you.
- If the provider asks about alcohol use, respond that you do not drink “because of your parents”.
  - If the provider asks an open-ended follow-up question, you can disclose that both of your parents “drank a lot and would sometimes hit you.”
- If the provider asks about safety in your current relationships, or if you feel safe at home, respond with “yes…mostly”.
  - If the provider asks a follow-up question, you can disclose that your partner sometimes hits you, but that mostly they are very kind to you and that you are engaged. Express concern that the provider will judge you for marrying someone that has hurt you.
- If the provider offers you resources, decline by stating that you feel safe and don’t need any help.

**Frequently asked questions:**

- *Do I need to call the police?*
  - No, the patient in this scenario is an adult (aged 18-65) and as such has a right to self-determination. Concerns indicating thorough follow-up would be present if there were children (under 18), elders (over 65), or persons with disabilities present in the home where the abuse is taking place. If you have safety concerns, always notify your precepting physician.
- *What can I do to help Ming realize they are unsafe and leave?*
  - While it can be upsetting, or even triggering to hear of Ming’s situation and choices, it is not helpful for providers to take the stance of convincing or pressuring patients to leave someone who is hurting them. Support, validation of the complexities of emotions, and understanding the conditions present that may deem it necessary or important to stay are important first steps. Offering resources as is done in this scenario is important. It is equally important to build unconditional trust so that if and when the patient is ready, they can talk to you about their choices.

**Guidance and Questions for Facilitator: Case 1**

The first case is written gender neutral - both the gender identity of the patient and the gender identity of their partner is not explicitly stated. If the students have questions, let them know they are able to choose the gender identity of the patient. The student playing the patient can choose to inform the student playing the provider what their gender identity is before the role play begins or if it comes up during the role play. The take home point is that IPV is prevalent and we want to avoid anchoring our minds to the idea that only women of childbearing age or only individuals in heterosexual relationships experience IPV.

Questions for the group:

How did it feel to ask/be asked about trauma?

For the student playing the physician:

1. Help the student who is the physician explore any sense of frustration/helplessness they may feel.
2. How did you as the provider address **safety**?
3. To address **Empowerment, Voice and Choice**:
   1. How can the provider act next to build on the patient’s current strengths?
   2. What choices can the provider offer now?
   3. How can the provider name the resilience or strength of the patient in today’s session?

For the student playing the patient:

1. Help the student explore what they felt like they needed from the provider in this session. Did they get what they needed? Did the provider try to understand your choice?
2. To address **Cultural, Historic and Gender Issues**:
   1. How did the patient feel their experience was responded to by the provider? Did they feel understood?
3. To address **Empowerment, Voice and Choice**:
   1. Did you feel like the provider built on your strengths?
   2. Did the provider make you feel like you had choices in the session?
   3. Did the provider name your strength/resilience?

**Teaching points**

- When patients are in abusive relationships, it can be distressing to know that when they leave clinic, they are likely to be entering back into an unsafe environment. As the provider, our immediate reaction is often to get them to what we feel is a safer situation as soon as possible. However, it is important to align yourself with the patient and meet them when they are at. Caring for patients experiencing IPV is done over time and not in one clinic visit.
- The patient is living with deeply traumatic experiences and is seeking medical care, despite worries that they may be judged for their history. Coming into clinic and seeking care is something we can always validate our patients for and name as a strength.
- When a patient discloses this history, it is important to remember that you are not the only member of their care team. As a medical student, it is important to notify your preceptor. If you know of local and clinic-based resources, you could offer those to your patient.

**Case 2:** Prepared by Taylor Brown, HMS ’21

**Provider Perspective:**

You are a medical student in the emergency department caring for Dominic a 25-year-old male (he/him/his) with a history of alcohol use disorder in remission, chronic pain, and chronic pancreatitis. He is presenting to the ED with an acute flare of his chronic pancreatitis. His urine toxicology screen is positive for oxycodone and a serum EtOH is undetectable. Prior to meeting him, you see that he has had multiple admissions to a community hospital emergency department for pancreatitis, and several of the notes express concern for “drug seeking behavior”. In the exam room, you encounter a young man writhing in excruciating pain. He is accompanied by his parents who mention that he has had bad experiences with other providers in the past.

*(One of the cornerstones for the standard of care for acute pancreatitis is adequately controlling your patient’s pain)*

Your objective is to obtain the HPI. Be sure to ask about pain and pain control.

**Patient Perspective:**

You are Dominic, a 25 male (he/his/him). You have a history of alcohol use disorder, now in remission. You have been abstinent from alcohol for the past three years. You live with your supportive parents and work from home as a graphic designer. When you were still using alcohol, you developed chronic pancreatitis, which has left you struggling with chronic pain for which you take daily medication (oxycodone). You are hospitalized at least once per month for uncontrolled pain and pancreatitis flares. At your community hospital, you are known to the emergency room physicians, who think you may also struggle with an opioid use disorder based on your many presentations for breakthrough pain and history of alcohol use disorder. You are frustrated with your treatment. During your last hospitalization, they attempted to place a thoracic nerve block to control your pain. Before the procedure began, they attempted to anesthetize you, but when the procedure began, you could still feel everything. You asked the physicians to stop, but they continued on with the procedure despite your repeated declarations of pain. Since this event, you have nightmares and flashbacks to the procedure. You have not been to a hospital since, but your parents have brought you to a different emergency department because you are very ill and have not stopped vomiting for two days.

The provider’s objective for this conversation is to collect the HPI.

- Initially, you will be hostile towards the provider and express that you are in excruciating pain.
- Answer HPI questions tersely, reminding the provider about your pain.
  - “When did the pain begin?”, respond “2 days ago”
  - “Has anything made it better or worse?”, respond “No”
- When the provider asks about your pain- tell the story about the doctors that did not listen when you told them to stop a procedure.
- If the provider asks follow-up questions about the event, do not make eye contact and stop responding.
  - If the provider asks what is wrong, state that you hate re-telling the story.
- If the provider offers your resources, respond that you would like to talk to someone about the experience since you keep having flashbacks.

**Frequently asked questions:**

- *What do I do when patients lie to me? While this patient didn’t, I fear others will, and I am especially concerned when addiction is a presenting issue*.
  - Regardless of the patient’s history or truth telling, addressing pain is always a critical part of the treatment plan. Provider culture regarding substance use can vary by setting, and it is not uncommon for there to be a pejorative tone regarding treatment of those addicted to substances. TIC requires a different approach, one of seeking to understand that patients are choosing their own path, and that support and the use of the six principles of TIC are the best path forward for meaningful treatment. It is not the job of a provider to enforce or coerce recovery, or seek to change presenting behaviors/attitudes of a patient, even if the patient is lying.
- *If a patient is silent, hostile or demeaning to me, what should I do?*
  - A trauma-informed approach does not mean allowing any and all patient statements or behaviors. Using clear communication that includes transparency can be an important response.
  - If the patient is unresponsive verbally but awake and alert, “*I respect your right not to answer me, and I know that I need to earn your trust. I will be transparent with you at each step of care.*”
  - If a patient is demeaning you can say: “*I understand that this may be a difficult time for you, however, the contact for care between us is important. I will do my very best to be respectful of you, and I require that you do the same for me. We will treat your concerns best if we work together to address your current needs and find a care plan that works for you.”*
  - If a patient threatens you, you can say: *“You just stated to me ____. That language is not accepted in this environment. I want to let you know that if you say this again to me or anyone here the next step is asking you to leave (or other policy of your environment).”*

**Guidance and Questions for facilitators:**

Questions for the group:

How did it feel to ask/be asked about trauma?

What types of trauma did this patient experience?

How have the patient’s past experiences influenced their perception of safety in the hospital?

For the student playing the provider:

What were your initial reactions to the patient’s history? Did you make any assumptions about this patient?

1. To address **Trust & Transparency:**
   1. Did you feel influenced by the notes from the community hospital? What were you concerned about before entering the patient’s room?
   2. Was it easy or difficult to elicit/listen to the patient’s perspective? Why or why not?
2. To address **Safety:**
   1. As the provider, how did you make the patient feel safe?
3. To address **Empowerment, Voice & Choice:**
   1. How can you empower the patient (and build trust), knowing the patient is distrustful of the medical system and learning that the patient experienced trauma from a medical procedure?

For the student playing the patient:

1. To address **Trust & Transparency:**
   1. Did you feel judged by the provider? Did you feel as though they were withholding information from you?
2. To address **Safety:**
   1. What did the provider do to make you feel safe and listened to in this scenario? If you didn’t feel safe or listened to, why?
3. To address **Empowerment, Voice & Choice:**
   1. Did you feel as though you had any control in this interaction?

**Teaching points**

- We are often biased by documentation in the medical record. It is important to read and gather data from available sources. However, it is critical to approach all patients with an open mind and without preconceived notions. Documentation is often biased and its permanence perpetuates these biases, particularly among patients with a history of substance use disorder.
- Safety is often established prior to communication with nonverbal cues - open body posture, maintaining eye contact, being at eye level. During conversation, giving patients space to tell their story and listening with minimal interruptions can also establish safety.
- Medical care and experiencing illness can be traumatic. Naming the patient’s experience can empower them and build trust. To validate them, you might say, “*The medical system earned your distrust by continuing a procedure when you clearly said no*.” To re-build trust, you may also say, “*My intent is to listen to you and make decisions with you*,” or, “*I hope that together we come up with a care plan that works for you*.”
